# Burnout and Perceived Stress of Polish Emergency Call Takers and Dispatchers

**DOI:** 10.3390/ijerph181910206

**Published:** 2021-09-28

**Authors:** Marta Makara-Studzińska, Maciej Załuski, Katarzyna Adamczyk

**Affiliations:** Division of Health Psychology, Faculty of Health Sciences, Collegium Medicum Jagiellonian University, 31-008 Cracow, Poland; marta.makara-studzinska@uj.edu.pl (M.M.-S.); katarzyna.1.adamczyk@uj.edu.pl (K.A.)

**Keywords:** emergency call-taker and dispatcher, perceived stress, occupational burnout, self-efficacy

## Abstract

A scientific research has demonstrated that emergency call operators face unique risks to job stress and burnout. It was hypothesized that perceived stress (demonstrated as resourcefulness–helplessness dimension) may mediate relationships between work environments and burnout taking into account the buffering effect of self-efficacy. The participants of the study were 546 emergency dispatchers and call-takers from 14 Polish public-safety answering points. The Link Burnout Questionnaire, the Perceived Stress Scale and the Generalized Self-Efficacy Scale were employed. The method of path analysis was used and direct and indirect interactions between the variables were identified. Shorter work experience (fewer years on this specific job position) was associated with a higher level of burnout. The greater number of shifts per month was associated with a higher level of perceived stress (higher level of helplessness). Self-efficacy was combined with perceived stress by antagonistic relationships, but the assumed buffering effect on burnout was not confirmed. It was observed that engaging the resource of one’s own self-efficacy in professional work may lead to the loss of other personal resources, manifesting itself in the form of greater disappointment with the work performed.

## 1. Introduction

Emergency call-takers and dispatchers (ECDs) are a key component of emergency care. The work of a dispatcher consists of receiving emergency reports from callers, registering them in the ICT system, collecting information on the type of event, its place and the number of injured persons, and then forwarding or redirecting the report to the appropriate dispatchers of rescue entities (medical rescue, fire service, police). The emergency notification system operating in Poland consists of 17 centers (public-safety answering point, PSAP), which form a uniform system for handling emergency notifications. In CPR in Poland, work takes place in a shift system equivalent to 12 h a day, from 7 a.m. to 7 p.m., and from 7 p.m. to 7 a.m. In Poland and in other countries, this profession has a high turnover rate. One of the reasons is that work exposes the employee to a number of specific stressors [1]. They include high responsibility for the safety of individuals reporting the risk and for the health of the dispatched personnel. Research carried out in one of the American ECDs center showed that 42% of operators assess their work as “stressful and very stressful”, 47% “demanding” and 14% “extreme demanding”, regardless of gender and length of service [1]. ECDs need to quickly identify specific indicator constellations by gathering critical information and effectively communicating appropriate first aid instructions. Communication difficulties resulting from the mental state of the callers who often provide insufficient information are a source of considerable psychological burden. Meanwhile, the health and life of another person may depend on the correct selection and assessment of the information obtained [2,3]. Despite the fact that the contact of the emergency number operator with traumatic stressors is made indirectly, by phone, scientists prove that it is as emotional as direct contact [4,5]. Conversation with a traumatized person may give ECDs symptoms of peritraumatic stress, secondary traumatic stress (STS), PTSD and other anxiety disorders, as well as depressive symptoms [6,7]. They are a consequence of i.e., empathetic and committed listening to traumatic content. It is not uncommon for callers to use verbal aggression or use obscene words, which also increases the stress of the operator’s work [8]. Symptoms of the burnout syndrome are indicated as the main cause of low retention in operator’s positions and high sickness absence [1]. ECDs report lower job and life satisfaction compared to employees working in other professions characterized by a high level of occupational stress. It is also noted that the causes of negative changes are not only working conditions, but also personal characteristics of some operators. Excessive involvement in work, ways of coping with work stress which are exhausting for psychophysical strength were indicated as predictors of work stress [1]. Risk factors are also: young age, female gender, lower education and lack of social and family support [5]. The ECDs’ work environment also exposes them to somatic health problems. This professional group is diagnosed with diseases such as obesity, frequent headaches, back pain and sleep disorders [8]. Shift work is not conducive to physical activity, which may protect against back pain and obesity especially if it takes place at night. Poorly organized breaks at work can contribute to faster fatigue of employees, additionally insufficient lighting of the workplace and high noise levels go hand in hand with maintaining high levels of cortisol in the blood. As in other professions, various environmental and individual factors can buffer the negative effects of stress. Lifestyle factors such as physical activity, active hobbies and emotional regulation skills can reduce the impact of occupational stressors. Personal characteristics such as self-efficacy, resilience, empathy and social support can contribute to the reduction of work-related stress. [6]. 

Burnout is explained with the help of theories linking stress at work resulting from the imbalance between work requirements and personal resources of an employee with psychophysical stress [9]. The state of psychophysical imbalance and tension prompts the employee to take defensive actions. Their presence is understood to be symptoms of the burnout process. The job demands–resources (JD-R) model [10] is one of several models explaining the mechanisms of occupational burnout and work commitment. Professional work engages energy levels, which are responsible for experienced a sense of energetic and effective connection with work, which may lead to a depletion of psychophysical resources and the deterioration of health. Energy processes are modified by the motivational processes related to the possessed resources (e.g., self-efficacy), which protect against the development of burnout. By using problem-focused coping strategies, beliefs that it is possible to meet demands and a belief in good outcomes in life, committed employees avoid burnout [11]. Occupational burnout syndrome is a result of the depletion of mental and psychical energy as well as the cognitive resources of a person [12]. The symptoms of occupational burnout develop as a consequence of an overload caused by workplace requirements until a person’s psychophysical resources are depleted, which in effect decreases their motivation to engage with their work. According to the assumptions of the Conservation of Resources (COR) model, burnout is the result of actions taken by an employee motivated by repeated loss or the threat of losing their personal resources [13]. The accompanying stress can contribute to the development of burnout, especially when resources are lost despite attempts to preserve them. According to the most recent definition that will come into force in January 2022, burnout is a syndrome conceptualized as resulting from chronic workplace stress that has not been successfully managed. It is characterized by three dimensions: feelings of energy depletion or exhaustion; increased mental distance from one’s job, or feelings of negativism or cynicism related to one’s job; and reduced professional efficacy [14]. Work-related stress belongs to the broad group of psychological risks, which have the potential to cause psychological and physical issues [15]. Cox [16] distinguished 10 categories in order to characterize sources of work-stress in the social and organizational context of work. Among the potential sources of health risks are mentioned: combined exposure to physical and psychosocial risk, job insecurity, high emotional load related to burnout and others [17]. 

Apart from stress understood as a process of physiological changes preparing the body to fight or flight responses, stress is understood as a psychological process. It is a result of the transaction between the individual and the environment, when environment demands are evaluated as exceeding the individual’s resources, especially in personally significant situations [18]. Considering stress at work solely from the perspective of physiological changes implies that the conditions of the work environment themselves are the cause of psychophysiological tension and occupational burnout. Meanwhile, the employee actively interacts with his work environment and the events that occur in his life. The cause of psychological stress is not the situation itself, but the emotional response to the situation mediated by the influence of cognitive processes [19]. The impact of stressful events is, to some degree, determined by one’s perception of their stressfulness, in categories of their predictability, control and sources of overload [18]. Perceived stress is an outcome variable, measuring the experienced level of stress as a function of objective stressful events, coping processes, personality factors [19] (p. 386). Individuals with a high level of perceived stress considered their lives as unpredictable, uncontrollable and overloading [op, cit, p. 387]. Psychological stress is influenced by both the objective features of a situation (i.e., work requires constant cognitive, emotional, or physical effort) and dispositional variables, including the aforementioned sense of self-efficacy and a subjective evaluation of efforts undertaken during struggles with daily adversities. Results from some studies show the correlation of evaluation with the respondent’s sex and age [20]. There are correlations between the level of perceived stress and the number of critical live events in a group of younger people and lack of such a relationship in a group of older people [20]. The studied age groups may differ in the role that different life events play role in the assessment of perceived stress in relation to age-specific expectations and chronic stressors. There are few studies that have explored the relationship between the perceived stress, which precedes the physiological response and others psychological processes. Research has shown that perceived stress can lower the efficiency of some cognitive functions [21]. The acceptance of the psychological stress variable into the burnout study allows us to see the role of cognitive assessment in the relationship between objective stressors and burnout. Predictors of stress at work of ECD are: young age, female gender, lower level of education and lack of social and family support [5]. Personal resources, understood as a person’s traits or state of mind, including their convictions on having control and influence over their work environment, play an important role in the phenomenon described above. Xanthopoulou et al. [11] researched the role of three personal resources in predicting occupational burnout and changes in work commitment: generalized self-efficacy, self-esteem and optimism. Self-efficacy is defined as the belief of an individual regarding the possibility to achieve an intended goal in a specific life situation [22,23]. According to the authors of the construct, people tend to regulate their level and distribution of effort according to the effects they believe their actions have on the effort. Employees’ beliefs about their own effectiveness are essential for their own perception about the context in which they work, especially when having to cope with highly demanding and potentially stressful job requirements [24,25]. In such cases, employees with a positive outlook on their own effectiveness adapt better to stressors at work [26]. On the contrary, those workers who consider themselves ineffective attribute failures to their competency deficit, thereby increasing the feeling of ineffectiveness [27]. Researchers noticed that personal resources partially mediated the relationship between work resources and commitment to work, protecting from burnout. A meta-analysis of studies conducted in various professional groups showed that, the average effect size estimate for the association between self-efficacy and burnout was a medium size (−0.33). Regarding the three burnout components, the largest estimate of the average effect size (−0.49) was found for the association between self-efficacy and lack of accomplishment [28]. Therefore, it is believed that positive self-esteem can alleviate the negative effect of work environment demands [29].

**Figure 1:**
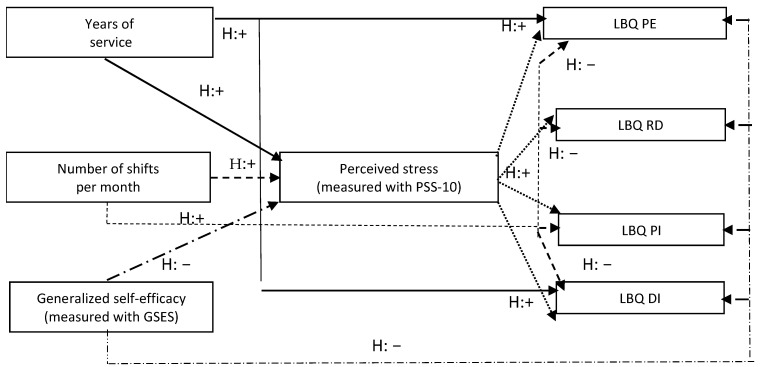
A proposed model of mutual links between experiences relating to ECDs’ work in the study on occupational burnout among Polish ECDs. GSES—General Self-Efficacy Scale, PSS-10—Perceived Stress Scale, LBQ—Link Burnout Questionnaire: PE—psychophysical exhaustion, RD—relationship deterioration, PI—sense of professional inefficacy, DI—disillusion.

In the Conservation of Resources Theory (COR) [30] the source of stress is the loss of resources needed for survival and no increase in new resources for survival. Stress is understood as a system of adaptive processes guiding the protection of resources. According to the theory, resources are directly related to each other, they form “caravans of resources”. The loss of one of them may lead to a spiral of subsequent losses. Generalized sense of self-efficacy, energy resources, sense of professional effectiveness and job satisfaction can be understood as interrelated human resources occurring in the professional work environment [30]. The information presented above became the basis for the research project presented in the article. Its purpose was to test the hypotheses on the role of the combination of personal resources among emergency number operators: the level of generalized self-efficacy, the level of perceived stress and factors related to the work performed: number of years of service, number of shifts per month in explaining the causes of occupational burnout. A hypothetical model of interrelationships (Figure 1) was developed to investigate the effect of these factors on burnout mediated by the level of perceived stress. 

The model assumes that the length of the dispatcher’s and call-taker’s work experience, the number of shifts per month and the level of perceived stress will have an impact on the increase in four aspects of occupational burnout. In other words, workers with shorter employment histories (usually younger in age, with less work experience, idealistic at work) will have a higher level of perceived stress and a higher level of burnout. Employees with a shorter professional experience go through the period of initial adaptation to the profession, then they verify their personal predispositions, develop mechanisms of coping with stress, adaptive ways of assessing occurring events. It is known from the literature that people with short work experience may be more exposed to the risk of burnout, which is caused by their maximum commitment and still undeveloped methods of coping with work stress [31]. However, not all studies support this [32,33]. Similarly, we assume that ECDs with more shifts per month will have higher levels of perceived stress and burnout. A greater number of shifts means a shorter time of returning to the energy and psychological balance and a greater number of interventions, not always successful. Knowing about the lack of developed, organizational methods of prevention against stress and burnout in the Polish PSAP system, we assume that the above assumptions are correct. On the other hand, the level of generalized self-efficacy will have an inversely proportional effect on both perceived stress levels and four aspects of burnout. 

## 2. Materials and Methods

### 2.1. Aim of the Study

The following research hypotheses were formulated:

**Hypotheses** **1a.** *Differences in seniority are responsible for the differences in the degree of occupational burnout in the group of ECDs. People with shorter work history (fewer years on the job in general) are exposed to a greater risk of burnout.*

**Hypotheses** **1b.** *Differences in the number of on-call shifts per month are responsible for the differences in the degree of occupational burnout in the group of ECDs. People with a greater number of on-call shifts in a month are exposed to a greater risk of burnout.*

**Hypotheses** **2a.** *The relationship between seniority with the degree of occupational burnout is moderated by the level of perceived stress. The high level of perceived stress strengthens the relationship between the length of service with the risk of occupational burnout in each of its four dimensions.*

**Hypotheses** **2b.** *The relationship between the number of on-call shifts per month with the degree of occupational burnout is moderated by the level of perceived stress. The high level of perceived stress strengthens the relationship between the number of on-call hours per month with the risk of occupational burnout in each of its four dimensions.*

**Hypotheses** **3.** *Generalized self-efficacy lowers the degree of burnout by influencing the level of perceived stress. A strong belief in self-efficacy lowers the risk of burnout, weakening the impact of perceived stress on burnout.*

**Hypotheses** **4.** *A low level of self-efficacy is associated with a high level of stress.*

The study also took into account the influence of controlled variables. These included: gender, age, level of education and having an actively cultivated hobby.

### 2.2. Procedure

The data for this study were collected between January and May 2020 among emergency call operators working in PSAP in Poland. The respondents completed an anonymous set of questionnaires which were mailed to their workplace. In total, 800 sets of research tools consisting of information about the study, three standardized research questionnaires, a demographic survey, and an invitation to participate were sent. Participation in the study was voluntary. After choosing appropriate subgroups participant completed sets of questionnaires. In total, 558 sets of questionnaires were returned, of which 546 were correctly completed. The questionnaires came from call-takers and dispatchers working in 14 out of 17 PSAP in Poland. 

### 2.3. Participants

The study participants were 546 emergency dispatchers and call-takers from 14 public-safety answering points (PSAP); consisting of 238 male (43.6%) and 308 (56.40%) female. The mean age was 34.37 (SD = 8.14; min = 19.0, max = 65.0), 232 (42.5%) of the participants were married, 143 (26.2%) cohabited with a partner, 120 (22%) single, 44 (8%) divorced, 3 (0.6%) widowed and 8 (0.7%) of them marital status was unknown. With regards to education, 396 (72.5%) of the ECDs had bachelor’s and master’s degree, 143 (26.2%) had secondary education, 1 (0.18%) vocational education and the level of education of 6 (1.1%) participants was unknown. The duration of service of the emergency call operators was: 0–2 years 148 (27.1%) participants, 3–4 years 131 (24%), 5–7 years, 199 (35%) and >7 years 63 (11.5%). Status of five (0.9%) participants was unknown. In regard to number of shifts in month, up to 13 shifts was seen in 63 (11.5%) participants, 14–15 shifts in 379 (69.4%), >15 shifts in 104 (19.1%). The “years of service” subgroups were set at the research planning stage. Participants were assigned to one of four followings groups: a short duration of service (0–2 years), a medium duration of service (3–4 years), a long duration of service (5–7 years) and a very long duration of service (>7 years). The groups had varying numbers of participants due to missing data (see Table 1).

The length of work experience correlates with the age of the respondents. People with up to 2 years of work experience have a lower level of education. In the groups of people with longer work experience, the percentage of married people is growing, and the number of single people, living in free relationships and divorced people is decreasing. Table 2 presents the number of people in each group in three specific subgroups that were created on the basis of their number of shifts per month. As the majority of respondents reported working either 14 or 15 shifts per month, the creation of a group of four would make it small. For this reason, three subgroups were distinguished.

In the case of the division into the number of on-call shifts per month (see Table 2), differences related to the age of the respondents were observed. The ECDs with more than 15 shifts a month were younger in age (25–30 years) and at the same time it was the most numerous age group of operators (37%).

### 2.4. Methods and Statistics

#### 2.4.1. Burnout

The level of burnout was assessed using the Polish version of the Link Burnout Questionnaire (LBQ) created by the Laboratory of Psychological Tests of the Polish Psychological Society [34]. The measure consists of 24 items in relation to which the subject responds on a 6-point Likert scale (1—never, 2—rarely, 3—once (or more) during a month, 4—more or less once a week, 5—several times a week, 6—every day). The questionnaire has four subscales according to four dimensions of occupational burnout: psychophysical exhaustion (PE), relationship deterioration (RD), professional inefficacy (PI), and disappointment (DI). The PE is the dimension of an employee’s psychophysical resources. One end expresses a state of exhaustion, fatigue and a feeling of being under pressure, the other end is a state of activity and vital energy. RD describes the quality of interpersonal relations with service recipients (people communicating by phone). One end describes the treatment of service recipients as objectively, indifference, hostility towards them, and the other—commitment to relationships and individual treatment of each caller. PI is a dimension related to the assessment of professional competences. One end of the dimension is characterized by a sense of effectiveness in the work performed, efficiency in the pursued professional goals, the other—a sense of ineffectiveness and lack of work results. DI is the dimension of the employee’s existential expectations towards the work performed. There are people who treat helping as a mission to help others. However, contact with professional reality can be disappointing. One dimension describes passion, enthusiasm and job satisfaction, the opposite—disappointment and lack of enthusiasm. Each subscale measures a range between low (6 pts) and high (36 pts) severity. The LBQ includes four indicators: the higher the score, the greater the intensity of each dimension of burnout. A typical study uses the mean point values obtained by a group of test persons for each dimension as indicators of burnout. In the individual study, the numerical values are converted into a sten scale using the standards developed for selected professional groups. The Polish version of the LBQ questionnaire has good psychometric properties. The scale of DI (Cronbach’s α = 0.84) has the highest internal reliability and the scale pertaining to the PI (Cronbach’s α = 0.68) has the lowest internal reliability. In our research, Cronbach’s α for individuals ranged from PI = 0.628; RD = 0.689; PE = 0.845; DI = 0.859. 

#### 2.4.2. The Level of Perceived Stress

The Polish version of the Perceived Stress Scale (PSS-10) [35] was employed. The PSS-10 questionnaire is the most widely used psychological instrument for measuring the perception of the cognitive aspects of stress and coping-appraising the effectiveness of coping strategies. The PSS-10 is designed to measure the level of perceived stress in terms of unpredictability, lack of control, and overload [19,35]. Ten questions of PSS-10 identify the level of perceived stress as an indicator of the effectiveness of dealing with life events. The questions in the PSS-10 ask about feelings and thoughts over the past month to which the respondent answers on a 5-point Likert-type scale: 0—never, 1—almost never, 2—sometimes, 3—quite often, 4—very often. The overall raw result ranges between 0 and 40 pts. The PSS-10 includes one indicator: the higher the score, the greater the intensity of the perceived stress. The results also allow prediction of the physical and mental discomfort of the respondents. In the Polish version, the scale has obtained very good psychometric properties with a Cronbach’s α value of 0.86. The Cronbach’s α in our research was 0.883. 

#### 2.4.3. Self-Efficacy

To measure beliefs about self-efficacy in the group of emergency call operators, a Polish language version of the Generalized Self-Efficacy Scale (GSES) was used [36]. The questionnaire based on Bandura’s theory of social learning, measures the strength of the general belief of the examined person about the effectiveness of coping with difficult life situations and smaller, daily hassles. Self-confidence in the tool tests, together with skills and knowledge, favor better coping in everyday life. The GSES consists of 10 questions to which the participant responds on a 4-point Likert-type scale: 1—not, 2—probably not, 3—probably yes, 4—yes. The overall raw result scores were between 10 and 40 pts. The GSES includes one indicator: the higher the score is, the greater intensity of the generalized self-efficacy it indicates. Cronbach’s α in the Polish version was 0.85. In our studies, Cronbach’s α was 0.883.

#### 2.4.4. Demographic and Other Factors

The controlled factors: age, education, hobbies and marital status were studied by using additional questionnaires. The seniority is both continuous and categorical independent variable. Four subgroups of respondents were distinguished according to the length of service. The participants completed the questionnaire by choosing the appropriate subgroup short, medium, long and very long duration of service.

The authors used software environment for statistical computing R (version 4.0.5, The R Foundation for Statistical Computing, Vienna, Austria) for statistical analyses [37]. A significance level of 0.05 was adopted in the analysis. Correlations between quantitative variables were analyzed using the Spearman coefficient. The comparison of the values of qualitative variables in the groups was performed using the chi-square test (with Yates’ correction for 2 × 2 tables) or the Fisher’s exact test where low expected numbers appeared in the tables. The comparison of the values of quantitative variables in two groups was performed using the Mann–Whitney test. The comparison of the values of quantitative variables in three or more groups was performed using the Kruskal–Wallis test. Once statistically significant differences were detected, the Dunn–Bonferroni post-hoc test was performed to identify statistically significantly different groups. The path analysis was performed in the R program, version 4.0.5 with the lavaan package [38].

#### 2.4.5. Ethics

The study protocol was approved by the Bioethics Commission at Jagiellonian University Medical College (decision No. 1072.6120.23.2017) and was carried out in accordance with the recommendations of the APA Ethics Code. 

## 3. Results

### 3.1. Correlations between Variables

Table 3 presents the correlation between the length of service, the number of on-call shifts per month in 2020, the level of perceived stress, the sense of generalized self-efficacy, and individual aspects of occupational burnout: psychophysical exhaustion, deterioration of interpersonal relationships, a sense of loss of professional effectiveness, and disappointment with the work performed. The results show that the longer length of service of the emergency number operator significantly correlates with two aspects of occupational burnout-deterioration of interpersonal relations and disappointment in the work performed. The mentioned correlations, although weak, were statistically significant. There was no correlation between seniority and the level of perceived stress and self-efficacy. The number of shifts per month significantly correlated with the level of perceived stress (higher level of helplessness). The level of experienced stress was significantly and positively associated with the sense of loss of professional effectiveness. The results show that the variable generalized self-efficacy is an important factor that negatively correlates with the level of perceived stress and one of the aspects of burnout—the sense of loss of professional effectiveness and, surprisingly, positively with the level of job disappointment. Statistical analyses also showed that the length of service and the number of shifts per month differentiated the values of the studied variables. Since each of the variables used correlated significantly with at least two other variables, there was no reason to remove any of them from the model.

### 3.2. SEM Analyzis

**Figure 2:**
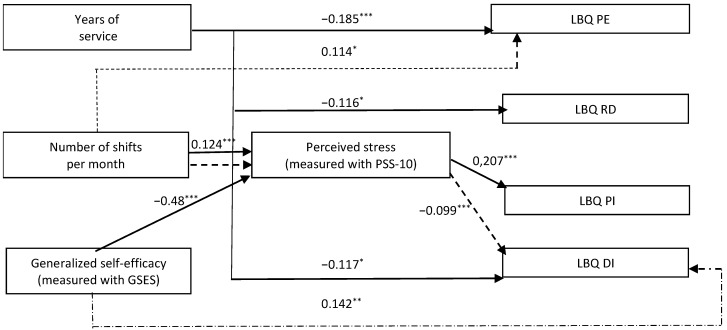
A model of path factors explaining various aspects of occupational burnout in a group of Polish ECDs. For the readability of the graph, the graphic markings of irrelevant paths were omitted. *** Statistical significance < 0.001; ** statistical significance < 0.01; * statistical significance < 0.05. GSES-GSES—General Self-Efficacy Scale, PSS-10—Perceived Stress Scale, LBQ—Link Burnout Questionnaire: PE—psychophysical exhaustion, RD—relationship deterioration, PI—sense of professional inefficacy, DI—disillusion.

The path SEM model presented in Figure 2 and Table 4 contains standardized results of the estimation of the strength of the relationship between the variables. As the tested model with mediation operates at degrees of freedom (df = 1), this gives rise to a very low RMSEA value and a very high CFI value. For this reason, it is not recommended to provide the value of measures of its fit (in our model RMSEA = 0; CFI = 1). Similarly, the chi-square statistic = 0 and has zero degrees of freedom.

A direct negative relationship was observed between the length of service and the level of psychophysical exhaustion and job disappointment. Shorter work experience coexisted with a higher level of psychophysical exhaustion and a higher level of disappointment with the work performed. This relationship also persisted after accounting for the indirect influence of the mediator-perceived stress. The level of self-effectiveness was positively associated with disappointment in the work performed, increasing his strength. People with higher self-efficacy levels reported greater disappointment in their work. Mediator—the level of perceived stress slightly weakened the strength of the aforementioned relationship. The level of perceived stress (as cognitive factor) was associated with a negative relationship with self-efficacy (a higher level of stress coexisted with a lower level of self-efficacy) and a positive relationship with the number of on-call visits a month (a greater number of on-calls was associated with a higher level of perceived stress-higher level of helplessness).

In two cases, the occurrence of a mediating effect of perceived stress on the relationship between independent variables and occupational burnout was observed. A greater number of shifts per month were negatively correlated with the level of job disappointment due to perceived stress. However, taking into account the direct impact of the number of shifts on the disappointment, the aforementioned effect lost its importance. Due to the level of perceived stress, longer length of service increased the negative relationship with the sense of loss of professional effectiveness. Additionally, in this case, taking into account the direct impact of seniority abolished the aforementioned effect. Taking into account the level of perceived stress weakened the positive relationship between self-efficacy and job disappointment, which still remained statistically significant. The mediator slightly weakened the positive relationship between the sense of self-efficacy and the disappointment in the work performed. The increase in the number of on-call duties per month after taking into account the indirect influence of the perceived stress coexisted with a higher level of psychophysical exhaustion. Taking into account the indirect impact of the perceived stress, the increase in the number of on-call duties coexisted with a higher level of psychophysical exhaustion. 

### 3.3. Additional Analysis and Results Including Controlled Variables

Comparing the level of perceived stress male and female ECD differences appeared between the sexes. Women reported significantly higher intensity of perceived stress (helplessness) compared to men. The stress level in the group of women was 1 point higher on the sten scale than in the group of men (6: 5 sten points). The results for both groups were in the middle range, the men’s scores were closer to the middle of the middle range (Me = 14), and the women’s scores were closer to the upper range (4–6 sten points, Me = 17). The comparison of burnout and perceived stress depends on the age of ECD using the Dunn–Bonferroni post-hoc test showed differences in one dimension of burnout and the level of perceived stress. Older workers (41 years and above) reported a significantly higher level of psychophysical exhaustion compared to younger people (25–35 years). The result of the elderly group was 7 sten (high score, Me = 22), whereas younger was 6 sten (upper limit of the mean score, Me = 21). Younger people (25–35 years) reported significantly higher levels of perceived stress than older people (41–45 years). The level of stress in the group of people aged 36–40 was within the range of 6 sten (Me = 17), people in the age range 41–45 was 5 sten (Me = 13). 

The comparison of burnout and perceived stress of ECD depends on the fact of having an active hobby, it showed significant differences (*p* < 0.001) only in the level of perceived stress. Participants with active hobbies reported significantly lower levels of perceived stress (5 sten, Me = 15) compared to those without (7 sten, Me = 20.5). It was also observed that people with hobbies prevailed in the group with shorter work history. Having an active hobby did not differentiate the respondents in terms of the dimensions of burnout.

## 4. Discussion

A model was tested assuming mediating the effect of the level of perceived stress on the relationships: seniority and number of on-call hours per month-self-efficacy-level of occupational burnout. The obtained results partially confirmed the assumption behind the hypothesis linking seniority with the risk of burnout (Hypothesis 1a). The study showed that fewer years on the job goes hand in hand with a higher level of psychophysical exhaustion, lowered the quality of interpersonal relationships and increased disappointment with the work performed. Among the respondents with a short period of work history, the youngest, less educated and living outside partnerships prevailed. The increase in seniority corresponded to the increase in the age of life, and thus also the number of married and better educated people increased. Short work history associated with a younger age of life was associated with greater psychophysical exhaustion of ECDs for three reasons. Firstly, people aged 25–35 are usually involved in a number of life goals at the same time: starting a professional career and becoming independent financially, starting a marriage and family, and further education. In the case of female, there were over 56% of them in the studied group, and the goal may also be the birth and care of the first child. A conglomerate of requirements is created that strongly engages human energy resources. Second, in this age range there are still many people who do not have a supportive partner. It is known from the literature that obtaining social support (from colleagues and family) is one of the important factors preventing the employee’s mental exhaustion [9]. The third reason concerns the human motivational processes. It is believed that people with relatively short work history are more likely to report signs of burnout due to a strong commitment to work due to idealistic job expectations. Their presence exposes them to a painful confrontation with professional reality [31,34]. The study by Smith et al. [39] showed that both the level of stress and the cause of the stress, i.e., the conflict of reconciliation between work, private, and family life, shaped the level of occupational burnout in the professional group of firefighters. Among the respondents with longer work experience and, at the same time, older in age, more people formed relationships and had university education. It should be noted that one of the reasons for disappointment with the work performed may be the fact that the Polish emergency notification system is in the process of being developed. It was only in 2020 that ECDs in Poland were covered by legal protection due to public employees. This is to prevent threatening ECDs with impunity by reporting persons. Regulations defined a method of promoting employees, the lack of which was demotivating for employees. The paths of professional development and promotion were determined by introducing gradation of positions. ECDs can move from the operator’s station to another managerial position: senior operator of emergency numbers, coordinator and coordinator–trainer of emergency operators. The obtained results are similar to those obtained in the survey among teachers [40]. Older workers with longer work history reported fewer burnout symptoms compared to younger workers. This was explained by the fact that the greater work history of older teachers allows them to cope better in various professional situations, while the younger ones experience a difficult period of adaptation to new professional situations. The opposite pattern was found in the study of doctors, where longer work experience and, at the same time, greater professional experience were associated with a higher level of perceived stress and a greater risk of burnout [41]. 

As for the assumed relationships between the number of on-call duties per month and the risk of burnout (Hypothesis 1b), the study confirmed them in the case of one dimension of burnout. The increase in the number of on-call duty in a month coexisted with a higher level of emotional exhaustion. The fact that the increase in the number of on-call duties leads to psychophysical exhaustion confirms the assumptions of COR theory. Human resources are interrelated in the system of the so-called “caravan of resources”, the loss of one (an increase in the number of on-call duties increases the depletion of energy resources) may entail further negative changes (feeling of psychophysical exhaustion). More on-call duty also means a greater risk of losing other human resources (e.g., sense of control and efficiency). The employee’s attempts to counteract the losses may additionally be psychophysically exhausting.

In the case of Hypothesis 2a, assuming that a high level of perceived stress mediates the relationship between seniority and the risk of occupational burnout, it was observed that the increase in the level of stress increased the sense of loss of professional effectiveness. Participants with greater job seniority reported a lack of effectiveness and lack of work results, when they were accompanied by a higher level of perceived stress. A higher level of stress means greater helplessness and less efficiency in coping with life adversities using the available coping strategy [19,42]. Similar results were obtained in a study of academics in Portugal [43]. The level of perceived stress was proportional to psychophysical exhaustion, and inversely proportional to the level of personal accomplishment. The weakest sense of personal accomplishment (measured with the MBI questionnaire) was reported among participants with the highest level of perceived stress, which would confirm the correctness of COR theory. 

In the case of Hypothesis 2b assuming that a high level of perceived stress mediates the number of on-call duties in a month and the risk of occupational burnout, the study confirmed them in terms of two dimensions of burnout, but in only one case, taking into account the assumed direction of dependence: an increase in the number of on-call duties coincided with an increase in the level of psychophysical exhaustion and with a lower level of job disappointment. Taking into account the role of perceived stress strengthened the positive relationship between the number of on-call duty hours and the level of emotional exhaustion. The obtained results show the combined influence of energy factors (greater requirements of the work environment related to a greater number of on-call duty) and psychological factors (negative assessment of the situation) on the depletion of the employee’s psychophysical resources. In the case of a lower level of disappointment with work, it should be assumed that the respondents on duty more often retained greater enthusiasm, passion and satisfaction with their work. It should be emphasized that disappointment with the work performed is a dimension that relates to the employee’s attitudes and existential expectations more than the conditions of the work performed. The research carried out in the group of Polish nurses showed that the level of dissatisfaction with work was low among women with strong beliefs about what is important in life [44]. When the nurse’s need to make life changes grew, it was accompanied by a loss of passion and enthusiasm related to professional activity. It is difficult to explain in this case the mediating role of perceived stress, where a high level of it is an indicator of a negative assessment of one’s competence in coping with life stress. The obtained results may hide complicated dependencies between the assessment of competences in coping with life stress, experiencing passion and enthusiasm while practicing the profession, and undertaking a larger number of shifts per month. In the JD-R model [10], the presence of physical tension in a work environment and its increase in intensity is understood as a manifestation of energy processes which, together with motivational processes, shape the level of work commitment. Engaged employees have a sense of energetic and effective connection with work, which they treat as a challenge. Difficulties arising at work do not cause overwhelming stress among them. They maintain a sense of vigor, dedication and absorption [45] (p. 74). In COR theory, stress is understood as an adaptive phenomenon that occurs in response to sequences of objective life events [30]. Stress serves to engage in activities focused on the protection and development of possessed resources. The maintenance and development of personal resources such as self-efficacy and low level of perceived stress protect against the occurrence of the burnout syndrome. It also prevents a spiral of resources loss. In the case of our research group, the increased level of perceived stress accompanying the work performed may result from both the objectively difficult conditions of the work environment, call takers and dispatchers in Poland, and the presence of events taking place in the non-professional life of the respondents.

In the case of Hypothesis 3, assuming that the belief about generalized self-efficacy is a buffer protecting the employee against burnout, weakening the mediating role of perceived stress, was not confirmed. First of all, the assumption that self-efficacy has negative relations with the dimensions of occupational burnout was only partially confirmed. We observed a negative relationship between self-efficacy and the professional inefficacy dimension. On the other hand, it was observed that a higher level of self-efficacy was accompanied by a greater sense of disappointment with the work performed in the respondents, which indicates a positive relationship between the variables. The obtained result is completely different from that obtained in the study of Polish nurses, teachers and firefighters, in which the sense of self-efficacy turned out to be a significant mediator changing the direction and strength of the relationship between the perceived stress and all four dimensions of occupational burnout [46,47,48]. In the case of these occupational groups, a strong negative correlation between the level of effectiveness and all 4 aspects of burnout was observed. In the transactional theory of stress by Lazarus and Folkman [49], the level of stress depends, among others, on the result of the secondary evaluation of the coping strategy. Thus, the belief in self-efficacy may constitute a resource that shapes the type of assessment of the situation in terms of stress [26,27]. This became apparent in the ECD’s study in the form of a strong negative relationship between effectiveness and perceived stress (Hypothesis 4). According to COR theory, the loss of resources has a strong negative impact on people, it quickly worsens and gives rise to a feeling of stress [11]. If the human situation does not allow them to invest in resources in order to protect themselves from losing them, this condition deepens. Currently in Poland, there are significant staff shortages in ECD’s positions, which goes hand in hand with an increase in the number of reports received by the operator during the on-call duty. There are shifts during which one ECDs receives up to 350 calls [50]. Due to work overload, ECDs in Poland work in the profession for 3–4 years on average [7]. The discussed dependencies: self-efficacy-disillusion describe the situation of a mismatch between the high sense of agency ECDs and the conditions of the work performed. According to COR theory, the depletion of personal resources may trigger a downward spiral of loss of other personal resources. People who engage in their work with a high sense of self-efficacy may experience more disappointment when faced with working conditions that risk losing other resources. In the examined group of ECDs, over 72% of the respondents had secondary and higher education, mainly technical and engineering, but also pedagogical, economic, philological and medical. It can be assumed that educational achievements were reflected in the high level of self-effectiveness of people from the studied group. Research aimed at capturing changes in the sense of self-efficacy due to the level of psychophysical exhaustion has shown that an increase in the level of exhaustion goes hand in hand with a decrease in the sense of self-efficacy, which in turn reduces the level of involvement in the work performed [51]. Moreover, the conducted meta-analysis of research on the relationships: self-efficacy-dimensions of occupational burnout revealed the strongest relationship in the case of the dimension of personal accomplishment [28]. It is believed that these concepts may refer to similar, difficult to distinguish variables that may even develop independently of each other [51]. In order to check the aforementioned dependencies, a longitudinal study model should be used, which allows to capture the dynamics of resource changes.

In the case of Hypothesis 4: assuming that a low level of self-efficacy is associated with a high level of stress, it has been confirmed. Both the correlation analysis and the path analysis revealed a negative relationship between the above-mentioned variables. This is probably due to the fact that both variables have a similar psychological basis: they relate to human beliefs about having an influence on the achieved goals, being effective in overcoming obstacles and solving problems. In the case of this hypothesis, the results turned out to be consistent with those obtained in many scientific studies [52]. The discussed dependencies draw attention to the role of cognitive factors on prevention of burnout in the researched professional group. The conditions of the working environment shape the level of burnout not directly, but indirectly through the level of perceived stress (resourcefulness-helplessness dimension). As already mentioned, workers with high level of perceived stress considered their lives as unpredictable, uncontrollable and overloading [12]. Perceived stress is an employee’s personal resource (cognitive factor) that can be shaped. This is especially important for employees with a short professional experience. It is equally important to strengthen the self-efficacy level of workers. Type of assessment of the work environment and one’s resources in terms of perceived stress triggers processes that can lead to loss of personal resources and burnout. However, in this case the dependencies are more complicated. Psychological assistance should take into account the length of service of workers, the number of shifts per month and personal resources including differences in the assessment of the work situation and the level of self-efficacy. The results of the study very clearly confirmed the importance of having an active hobby in protection against stress. On the other hand, a hobby does not directly prevent occupational burnout. 

## 5. Limitations of the Study 

The limitations of the study are seen in the cross-sectional design of the study. The burnout syndrome is a process that develops over time, and a longitudinal design might have yielded more results. One of the conditions for the assumption of mediation between the studied variables is to exclude the possibility that the relationship between the dependent variable and the mediator is inverse, that burnout causes a decrease in the sense of self-efficacy (risk of the error of reverse causality). One way to dispel doubts about the directionality of the relationship between the variables would be to conduct longitudinal studies. In the study it did not use objective methods that allow measurement of the medical parameters of burnout and stress, it and was conducted using only a self-report questionnaire. Self-report tools were used in the research, which is burdened with the risk of measurement errors (self-reported biases, social desirability). 

## 6. Conclusions

The results have shown that: (1) the level of perceived stress mediated relationships between work factors: number of shifts per month, seniority and particular aspects of occupational burnout. (2) Number of shifts per month increase perceived stress and psychophysical exhaustion. (3) Years of service increase the severity of some dimensions of burnout. (4) The correlation analysis confirmed the negative relationship between self-efficacy and the loss of professional effectiveness, and at the same time revealed a positive relationship between self-efficacy and disappointment in the profession. However, taking into account the mediating influence of perceived stress on the aforementioned relationships removed the aforementioned dependencies. (5) Engaging the resource of self-effectiveness in professional work may lead to the loss of other resources, manifesting itself in the form of greater disappointment with the work performed. (6) Complex relationships: perceived stress–self-efficacy burnout show the importance of taking advanced measures to prevent burnout. 

## Figures and Tables

**Table 1:** The group size of the Polish ECDs subgroups separated by the number of years of work.

Participants (N = 541)			
subgroup A (N = 148)	subgroup B (N = 131)	subgroup C (N = 199)	subgroup D (N = 63)

Subgroup A: up to 2 years of service; subgroup B: 3–4 years of service; subgroup C: 5–7 years of service; subgroup D: >7 years of service.

**Table 2:** The group size of the Polish ECDs subgroups separated according to number of shifts per month.

Participants (N = 546)		
Up to 13 shifts (N = 63)	14–15 shifts (N = 379)	Over 15 shifts (N = 104)

**Table 3:** Spearman’s correlations between the variables in a group of Polish ECDs (N = 546).

Variable	Spearman’s Correlations							
	Years of Service	No of Shifts per Month	PSS10	GSES	LBQ PE	LBQ RD	LBQ PI	LBQ DI
Years of service	---							
No of shifts per month	0.018	---						
PSS-10	0.039	0.133 **	---					
GSES	−0.016	−0.051	−0.537 ***	---				
LBQ PE	−0.075	0.02	0.031	0.03	---			
LBQ RD	−0.132 **	−0.03	0.049	0.033	0.353 ***	---		
LBQ PI	−0.043	−0.009	0.217 ***	−0.109 *	0.039	0.193 ***	---	
LBQ DI	−0.099 *	−0.007	−0.074	0.126 **	0.29 ***	0.239 ***	0.148 ***	---

*** Statistical significance < 0.001; ** statistical significance < 0.01; * statistical significance < 0.05. GSES-GSES—General Self-Efficacy Scale, PSS-10—Perceived Stress Scale, LBQ—Link Burnout Questionnaire: PE—psychophysical exhaustion, RD—relationship deterioration, PI—sense of professional inefficacy, DI—disillusion.

**Table 4:** Standardized results of regression analyses performed on the total effect, direct effect and indirect effect in group of Polish ECDs (N = 546).

Variable	Effect (β)											
	Generalized Self-Efficacy (Measured with GSES)			No of Shifts per Month			Years of Service			Perceived Stress (Measured with PSS-10)		
	Direct	Indirect	Total	Direct	Indirect	Total	Direct	Indirect	Total	Direct	Indirect	Total
Burnout												
PE	0.068	0.011	−0.049	0.052	0.002	0.114 *	−0.185 ***	0.005	−0.18 ***	0.088	---	0.088
RD	0.091	0.005	0.082	0.077	0.011	0.063	−0.116 *	0.003	−0.113 *	0.039	---	0.039
PI	0.025	−0.042	0.025	−0.008	0.008	−0.035	−0.06	0.026 *	0.018	0.207 ***	---	0.207 ***
DI	0.142 **	−0.031	0.111 *	−0.043	−0.099 ***	−0.074	−0.117 *	−0.018	0.073	0.064	---	0.064
PSS-10	−0.48 ***	---	−0.48 ***	0.124 ***	---	0.124 ***	0.052	---	0.052	---	---	---

*** Statistical significance < 0.001; ** Statistical significance < 0.01; * Statistical significance < 0.05; GSES—General Self-Efficacy Scale, LBQ—Link Burnout Questionnaire, PSS-10—Perceived Stress Scale. PE—psychophysical exhaustion, RD—relationship deterioration, PI—sense of professional inefficacy, DI—disillusion.

## Data Availability

The data presented in this study are available on request from the corresponding author. The data are not publicly available due to the privacy and professional specificity of the people who took part in this research and who work the Polish emergency system.
